# In Operando Optical Analysis of Electrolyte Colour Change and Its Correlation to Capacity Fade in Li-Ion Cells at Elevated Temperatures

**DOI:** 10.3390/s24237686

**Published:** 2024-11-30

**Authors:** Saud Sattar, Thomas Statheros, Ali Raza, Quirin Kellner, Yifei Yu, Rohit Bhagat, Alexander J. Roberts, Yue Guo

**Affiliations:** 1 Centre for E-Mobility and Clean Growth, Coventry University, Coventry CV1 5FB, UK; 2 Centre for Future Transport and Cities, Coventry University, Coventry CV1 5FB, UK; 3 FEV UK Ltd., Coventry CV1 2TL, UK; 4 State Key Laboratory of Material Processing and Die & Mould Technology, School of Materials Science and Engineering, Huazhong University of Science and Technology, Wuhan 430047, China

**Keywords:** electrolyte colour change, Li-ion cell, senor instrumentation, battery diagnostics

## Abstract

The existing body of research on battery state of health has identified various degradation modes for the electrolyte, yet very few studies have explored the role of electrolyte colour changes as a diagnostic tool for state of health (SOH). This study investigates the impact of elevated temperatures and its correlation with electrolyte colour changes and capacity fade during cycling. Specifically, the research examines whether cycling cells at elevated temperatures induces noticeable changes in electrolyte colour and whether these changes can be linked to the SOH of the cells. The methodology employs in operando optical sensors to monitor real-time colour shifts in the electrolyte, aiming to demonstrate a qualitative relationship between electrolyte colour change, degradation, thermal ageing, and capacity fade, laying the foundations for future quantitative assessment of the relationships identified. Our research builds upon these findings by offering a novel approach that integrates optical sensing to provide real-time visual evidence of electrolyte degradation and colour change during cell operation. The results demonstrate a clear relationship between elevated temperature, electrolyte colour change, and capacity fade, leading to accelerated degradation. This approach offers a new insight over traditional in exitu battery diagnostics, as it enables continuous in operando monitoring of electrolyte colour change and has the potential to unlock a detailed understanding of the chemical reactions and electrolyte breakdown during cycling.

## 1. Introduction

With the need to shift to an environmentally sustainable future, lithium-ion batteries (LiBs) are finding new roles to decarbonise a range of technologies and help achieve net zero. With their greater utilisation in a range of applications, ever-increasing demands are being placed on the lifetime of LiBs; in application, a greater understanding of the electrolyte performance and its electrode interaction is needed. Various degradation mechanisms are correlated to electrolytes and can significantly impact the performance and life of a cell [1,2]. Since the solid electrolyte interphase (SEI) layer consists of decomposed products of electrolyte solvent and additives, its characteristics are also based on the composition of the electrolyte [3,4]. To improve battery lifetime and safety, a greater understanding of electrolyte composition, cell degradation, and their effects on state of health is needed [5,6,7,8,9].

When LiBs operate at elevated temperatures, an increasing rate of electrolyte degradation produces gases and other compounds via side reactions and chemical reactions, increasing the internal resistance and causing accelerated capacity fade [3]. As the temperature of the cell exceeds 45 °C, organic components of the electrolyte start decomposing, causing the emission of gases such as CO_2_, which may result in increased internal pressure, leading to swelling of the battery housing and, in extreme cases, thermal runaway [5]. The reversible capacity is further decreased through lithium consumption and loss of lithium inventory consumption [6]. Recent studies have identified how the colour of the electrolyte of a cell changes as it ages [1,10,11,12]. The colour change in electrolytes can be attributed to several factors, including the decomposition of organic solvents, electrode degradation, and the formation of oxidation or reduction byproducts, particularly at elevated temperatures or during high C-rate charging [11]. These processes, such as the release of metal ions from electrodes or the degradation of additives, can result in visible colour shifts, which may serve as indicators of chemical reactions and ageing mechanisms such as electrolyte degradation, thermal degradation, and lithium plating [12].

The degradation and colour change of electrolytes in lithium-ion batteries is a complex process influenced by various factors beyond repetitive charging and discharging cycles, including extreme temperatures, elevated voltages, high C-rates, and contaminants, all of which can lead to gassing, swelling, chemical corrosion, and decreased safety and performance [11,13,14,15,16,17,18,19]. Studies have noted electrolyte colour changes as an understudied indicator of degradation, with temperature and charge cycles linked to these changes, suggesting potential for developing diagnostic tools to detect early signs of degradation. Further investigation into these phenomena is needed to enhance the electrolyte’s performance and improve diagnostic accuracy [11,12,20].

There is a need for a combination of techniques to fully understand and correlate the degradation of the electrolyte with colour change. Such techniques would typically include solution NMR, UV–VIS spectroscopy, and FTIR to enable an analysis of speciation in the electrolytes as degradation and colour change progress. Through this combinatorial approach, a full degradation reaction mechanism may be possible [21,22,23,24,25,26,27]. These techniques show that electrolyte colour change is an area of research which has great potential to provide a deeper understanding of electrolyte degradation and its relationship with the state of health (SOH) of a cell. Therefore, understanding the relationship between electrolyte colour change, elevated temperatures, and capacity fade is imperative to further understand and analyse these degradation mechanisms and to gain an in-depth understanding of electrolytes.

**Figure 1:**
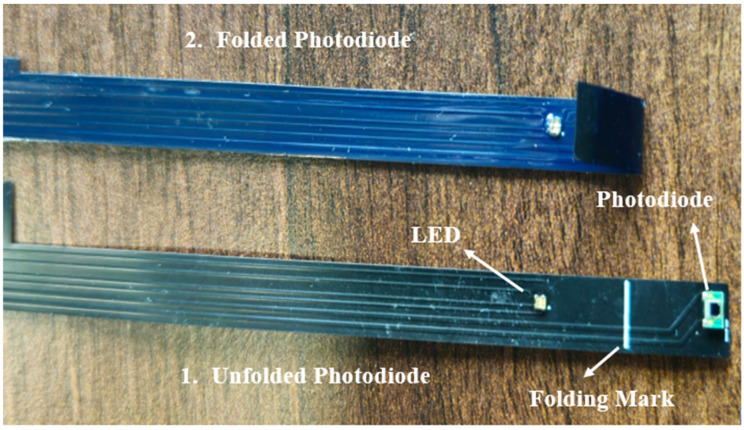
A foldable design of the photodiode and LED [2].

This study extends the concepts and methodologies developed in our previous work by advancing the understanding of the correlation between electrolyte colour and degradation, which is accomplished in real time through the instrumentation of a photodiode (S10993-02CT) and an RGB LED (150044M1552604) inside a pouch cell as shown in Figure 1 [1,10]. This approach allows us to delve deeper into the mechanisms of electrolyte discoloration, enhancing the insights gained from our previous work [10] and driving forward a more detailed understanding of electrolyte colour change. In this study, the degradation behaviour of electrolyte colour change in commercially available lithium-ion pouch cells is investigated by performing an accelerated ageing process, using high C-rate (2C) cycling and comparing their colour change at 25 °C, 40 °C, and 55 °C over 200 cycles in real time using an optical sensor. As a result of the targeted accelerated ageing of the cells, the electrolyte experiences degradation, which leads to observable changes in its colour.

## 2. Materials and Methods

This study uses 1 Ah dry lithium-ion pouch cells measuring 51 mm × 66 mm, manufactured by Li-Fun Technology, Zhuzhou City, China. These cells contain a nickel manganese cobalt oxide (NMC 532) cathode and a graphite anode and are supplied dry and sealed. They were filled with 4.0 g of electrolyte LiPF_6_ in EC (ethylene carbonate): EMC (ethyl methyl carbonate) 3:7 + 2% VC (vinylene carbonate), inside an argon-filled glove box, then vacuum sealed and soaked for 24 h at 25 °C. To help focus the discussion, the cells will be referred to in accordance with the temperature at which they were cycled, namely Cell25, Cell40, and Cell55.

For the sensor instrumentation of the cells, we employed a technique like the one utilised in our previous work for integrating optical sensors [10]. A polymer separator of a 0.60 mm width was placed in orientation between the LED and the photodiode, after which the sensors were folded in a direction where they were facing each other. This translucent separator, which is non-conductive, absorbs the electrolyte, thereby influencing the intensity of RGB light that penetrates depending on the colour of the electrolyte. The sensors were inserted on the outside of the multilayer stack within the cell form (with the same corresponding position in all cells), which was subsequently filled with electrolyte and sealed in the glove box.

The formation cycle of the pouch cells was performed using constant current constant voltage (CC-CV) cycling between a potential of 2.5 V to 4.2 V at a C-rate of C/20 with a CV cut-off current of C/100; this process was repeated for two cycles to ensure the formation of the SEI layer.

After formation, the cells underwent 200 cycles at constant temperatures of 25 °C, 40 °C, and 55 °C, where temperature-controlled chambers (Binder) maintained the desired temperatures. The cells were kept at equilibrium for six hours before cycling was initiated. The cells were cycled via CC-CV cycling between 2.5 V and 4.2 V at a C-rate of 2 C, with a CV cut-off limit of C/5.

The colour change of the electrolyte was recorded at the end of every cycle for a comparison of colour change between cycles. This study will involve a comparative analysis of the intensity of the red, green, and blue light at each of the three temperatures to determine their correlation with temperature and capacity fade.

## 3. Results and Discussions

### 3.1. Formation Cycles: Results and Analysis

**Figure 2:**
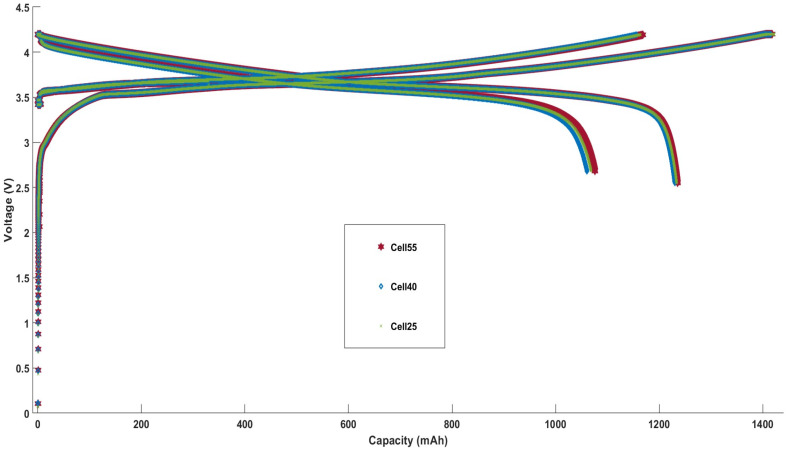
Formation profiles for Cell25, Cell40, and Cell55.

The formation cycle voltage profiles for Cell25, Cell40, and Cell55 are shown in Figure 2. An inspection of these profiles shows that the initial capacity change between each of the cells is within the cell-to-cell variation as shown in Figure 2. Cell capacities of ≈1070, ≈1065, and ≈1075 mAh were observed for Cell25, Cell40, and Cell55, respectively, with a first-cycle efficiency of ≈87% for each of the cells.

### 3.2. Comparative Analysis of Capacity Fade at Elevated Temperature

**Figure 3:**
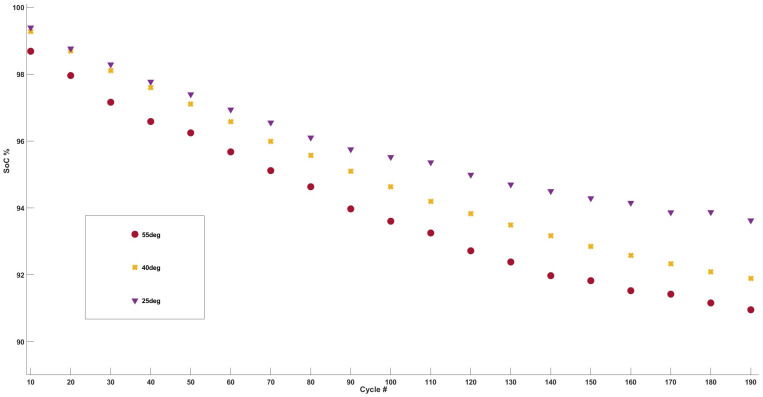
Capacity fade analysis for Cells 55, 40, and 25 vs cell cycle #.

Cell capacity over 200 cycles is shown in Figure 3, which shows an expected increase in the rate of capacity fade depending on the temperature of the cell.

The capacity drop in each cell is presented in Table 1. The table shows that, as expected, Cell55 has the highest drop in capacity (9%), followed by Cell40 (8%) and Cell25 (6%) over 200 cycles. It can also be seen that Cell55 and Cell40 had a 3% drop in the last 100 cycles, while Cell25 had a 2% drop for the same interval. This analysis indicates that the rate of capacity fade increases as the cell’s cycling temperature rises.

### 3.3. Electrolyte Colour Change over 200 Cycles

In this section, the bar graphs for each of the cells are shown where the successive columns in the background have been colour-coded with respect to the RGB colour of the electrolyte at the end of that cycle number. This colour-coding depicts the stage-by-stage progress in the colour shifts of the electrolytes during the testing period, hence determining how a change in the electrolyte colour relates to the capacity of the cell in relation to the number of cycles.

#### 3.3.1. Cell25

**Figure 4:**
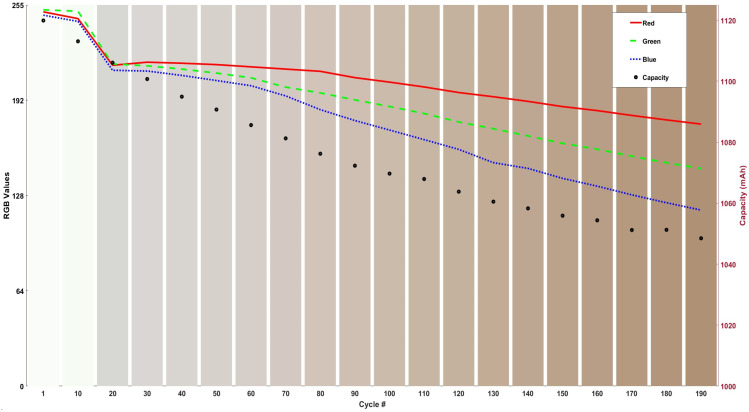
RGB values and capacity with the cycling number, with a visual representation of colours represented in the columns in the bar graph at 25 °C cycling.

The bar graph shown in Figure 4 shows the RGB colour change represented by vertical bars and the capacity fade of the cell with respect to the changes in the relative colour of the electrolyte.

Analysis of the bar graph reveals that the electrolyte begins to shift towards a brown hue around the 80th cycle, coinciding with fluctuations in the blue and green plots. By the 130th cycle, the electrolyte darkens considerably, corresponding to another fluctuation in the blue plot. These observations suggest that chemical/side reactions may have occurred within the cell at both the 80th and 140th cycles, altering the electrolyte’s colour to a darker shade. Table 2 presents the percentage change in both cell capacity and RGB colour intensities over 200 cycles.

Red intensity decreased by 30%, with an 18% reduction in the first 100 cycles and an additional 15% drop between cycles 100 and 200. Green intensity exhibited a significant overall decline of 42%, evenly distributed as a 24% reduction in each half of the cycling period. Blue intensity, showing the largest change, decreased by 53%, with 28% occurring in the first 100 cycles and a further 34% in the second 100 cycles, indicating a possible chemical reaction or species formation in the electrolyte that reflects blue wavelengths more than red.

#### 3.3.2. Cell40

**Figure 5:**
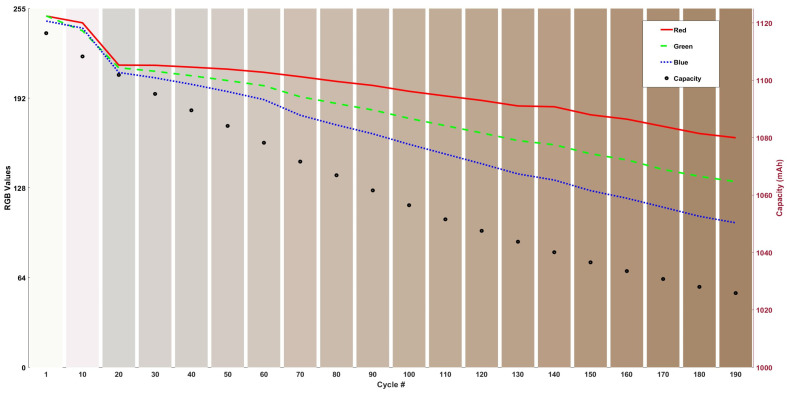
RGB values and capacity with the cycling number, with a visual representation of colours represented in the columns in the bar graph at 40 °C cycling.

The bar graph shown in Figure 5 shows the RGB colour change and capacity fade of the cell with respect to the changes in the relative colour of the electrolyte.

The bar graph in Figure 5 shows that, compared to Cell25, the RGB intensity for Cell40 decreased, which indicates that the electrolyte’s colour followed a similar colour change pattern, though at a 5% accelerated rate. Further visual analysis of the graph reveals that the electrolyte begins turning brown around the 70th cycle, with fluctuations in the blue and green plots, mirroring the behaviour observed at the 80th cycle in Cell25. The electrolyte of Cell40 becomes visibly darker around the 110th and again at the 140th cycle, suggesting that chemical/side reactions within the cell may have occurred at these points, altering the electrolyte to a darker shade. It is also visually evident that the overall colour of the electrolyte in Cell40 is darker than that of Cell25, paralleling the capacity fade trends observed in both cells. The percentage change analysis in capacity and RGB colours of the cell over 200 cycles is shown in Table 3.

Over 200 cycles, red intensity decreased by 35%, with most of the reduction occurring in the first 100 cycles (20%). Green intensity dropped by 47%, with a more pronounced decline in the second 100 cycles (28%). Blue intensity showed the most significant change, with a 58% reduction, primarily occurring in the second half of the cycle range.

#### 3.3.3. Cell55

**Figure 6:**
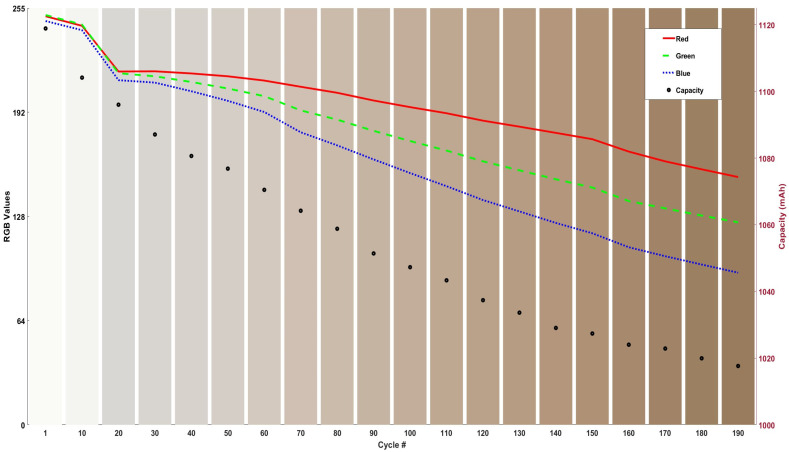
RGB values and capacity with the cycling number, with a visual representation of colours represented in the columns in the bar graph at 55 °C cycling.

The bar graph shown in Figure 6 shows the RGB colour change and capacity fade of the cell with respect to the changes in the relative colour of the electrolyte.

The bar graph in Figure 6 demonstrates that, like Cell25 and Cell40, Cell55 exhibited the largest change in blue intensity (62%) and the smallest change in red intensity (39%). However, compared to Cell40, the RGB intensities for Cell55 decreased by an additional 4% across all colours. This indicates that out of all the cells, Cell55 experienced the highest change in RGB colour intensity and the greatest capacity fade, suggesting it aged the most, as anticipated. Visual analysis further shows that Cell55 developed the darkest brown hue at the end of 200 cycles, compared to Cell40 and Cell25. The bar graph also reveals that the electrolyte began to turn brown around the 70th cycle, with fluctuations in the blue and green intensities, closely resembling the behaviour observed in Cell40. The electrolyte of Cell55 darkened significantly around the 150th cycle, accompanied by a sharp decrease in RGB intensities, suggesting chemical/side reactions or events may have occurred at this point, causing the electrolyte colour to change. The percentage change analysis in capacity and RGB colours of the cell over 200 cycles is shown in Table 4.

Over 200 cycles, red intensity decreased by 39%, with a 21% reduction in the first 100 cycles and an additional 24% in the following 100. Green intensity showed a total decline of 51%, with drops of 28% and 31% in the first and second 100 cycles, respectively. The blue intensity in Cell55 experienced the most significant change, decreasing by 62% over 200 cycles, with reductions of 34% in the first 100 cycles and 43% in the subsequent 100 cycles.

Overall, this analysis indicates that out of the three cells, Cell55 exhibited the largest decline in RGB intensities, corresponding to the most pronounced colour change and capacity fade among the cells, implying the highest degree of degradation. These results further confirm that as the cell cycles at elevated temperatures, the electrolyte darkens, paralleling capacity fade, and establishes a clear correlation between cycling temperature, electrolyte colour change, and capacity fade.

### 3.4. Comparison Between Individual Colour Intensity of Cells 55, 40, and 25

This comparative analysis aims to understand how each cell responds to changes in individual colour intensities, to assess any similarities or differences in colour evolution, and to further understand the earlier results from the bar graphs. Additionally, a differential analysis for each cell’s individual colour was carried out by plotting RGB values against cycle numbers, providing insight into the rate of colour change over intervals of 10 cycles.

#### 3.4.1. Blue Intensity Comparison

**Figure 7:**
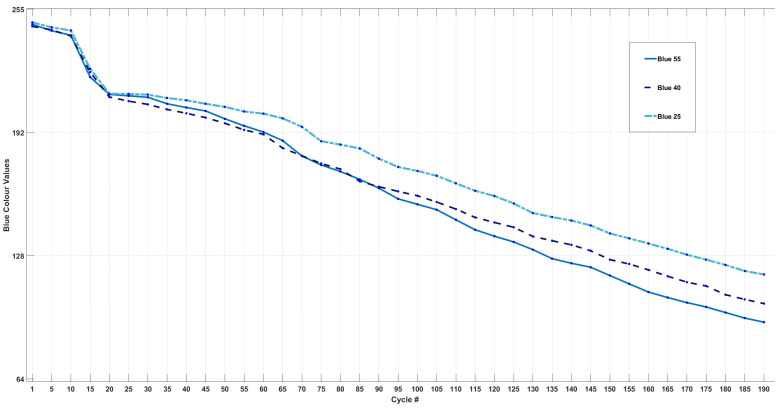
Change in blue LED intensity for Cell55, Cell40, and Cell25.

Figure 7 shows the trends of each cell’s change in blue intensity throughout 200 cycles.

The variations in the blue colour intensity for each cell are summarised in Table 5. In agreement with the findings in Section 3.3, Cell55 exhibited the largest decrease in blue intensity, recorded at 62% over 200 cycles. Furthermore, it is evident that each cell experienced a significantly greater change in blue intensity during cycles from 100 to 200, compared to cycles from 0 to 100. Specifically, Cell25 exhibited a 6% higher change, Cell40 showed a 5% higher change, and Cell55 demonstrated a 9% higher change in the latter 100 cycles relative to the first 100 cycles. This observation suggests that the chemical reactions or species formed between cycles 100 and 200 may reflect blue wavelengths more than those during the initial 100 cycles.

**Figure 8:**
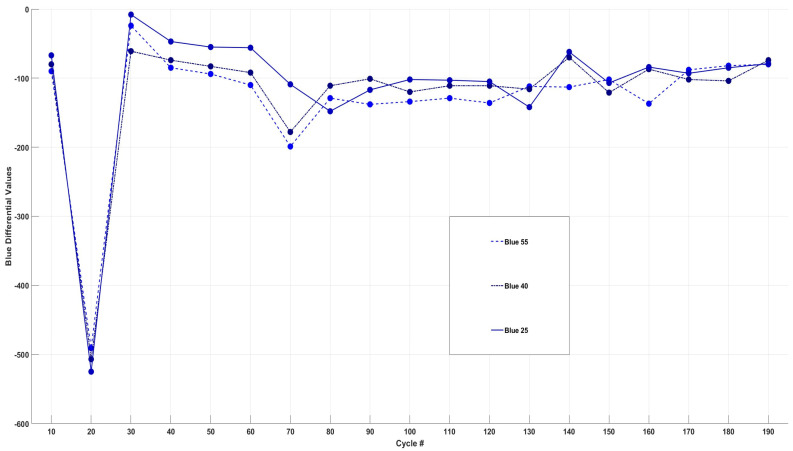
Differential analysis of change in blue intensity for Cell55, Cell40, and Cell25.

Figure 8 shows the differential analysis of each cell’s change in blue intensity throughout 200 cycles.

The differential analysis of blue intensity data, shown in Figure 7, reveals a significant decrease across all three cells at the 20th cycle. This decline corresponds with the bar graph results, marking the onset of colour change in the transparent electrolyte. Additionally, peaks in blue intensity were observed at cycle 70 for Cell40 and Cell55, and at cycle 80 for Cell25, consistent with the colour changes noted in Section 3.3. Similar patterns emerged at cycle 140 for Cell25 and Cell40, with a notable intensity drop at cycle 150 for Cell55, coinciding with the colour changes shown in Figure 5. Overall, blue intensity exhibited the most pronounced changes out of the three colours, particularly in the final 100 cycles, suggesting it plays a key role in electrolyte colour shifts and possibly indicating that the formation of any species or chemical/side reactions during cycling may be more reflective to blue wavelengths.

#### 3.4.2. Green Intensity Comparison

**Figure 9:**
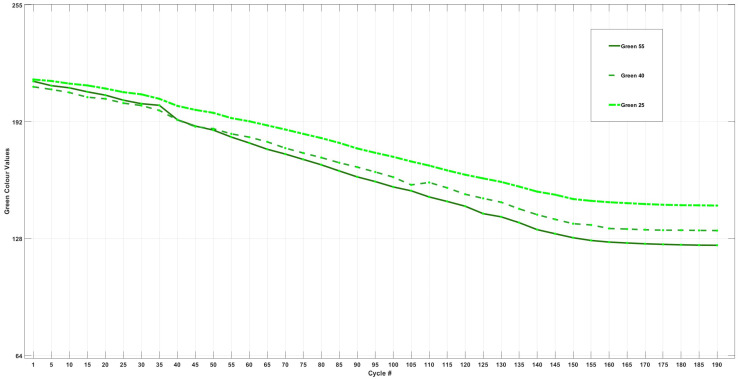
Change in green LED intensity for Cell55, Cell40, and Cell25.

Figure 9 shows the trends of each cell for the change in green intensity throughout 200 cycles.

The changes in green intensity for each cell are summarised in Table 6. As observed in the table, and like the trend observed in Section 3.4.1 (Table 5), Cell55 experienced the greatest loss in intensity, totalling 51% over 200 cycles. However, in contrast to the fluctuations depicted in Figure 7 for cycles 0–100 and 100–200, the cells exhibited a more uniform change in intensity throughout these intervals. This observation suggests that the chemical/side reactions or the formation of species related to their reflection to green wavelengths are significantly more uniform across the entire 200 cycles compared to the changes seen in blue intensity.

**Figure 10:**
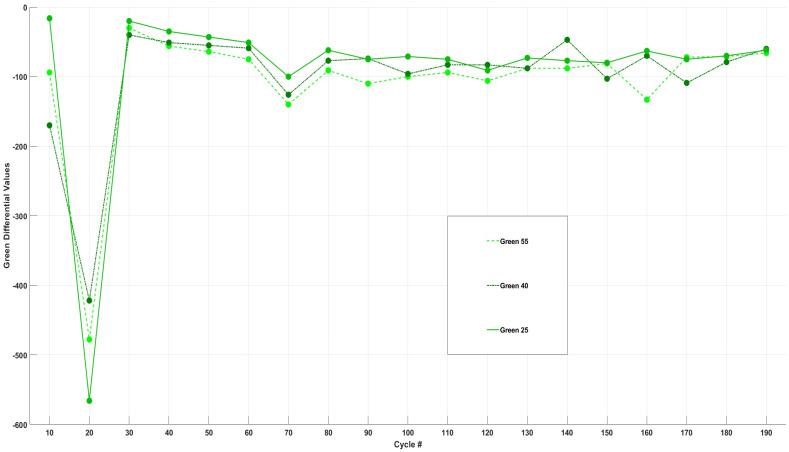
Differential analysis of change in green intensity for Cell55, Cell40, and Cell25 vs. cell cycle #.

Figure 10 shows the differential analysis of each cell’s change in green intensity throughout 200 cycles.

Analysis of the differential data for the green intensity of each cell presented in Figure 10 indicates a marked decline in green intensity across all three cells at the 20th cycle. When this finding is correlated with the bar graphs, it becomes evident that this cycle represents the onset of a colour change in the transparent electrolyte. Furthermore, peaks are observed at cycle 70 for all three cells, which can suggest the uniform formation of a species or chemical/side reactions that exhibit greater reflection to green wavelengths. Additional peaks are noted at cycle 160 for Cell55 and at cycle 170 for Cell40.

Overall, green intensity demonstrated a more consistent change across all three cells compared to the variations seen in blue intensity, as the change in green intensity over the course of 200 cycles was evenly distributed, with approximately 50% of the total change occurring in the first half and 50% in the second half, as opposed to the observations made in Figure 8 (blue), where the intensity dropped more during the last 100 cycles. This pattern suggests that the formation of any species and chemical/side reactions were reflective to green wavelengths and were relatively homogeneous throughout the cycling process.

#### 3.4.3. Red Intensity Comparison

**Figure 11:**
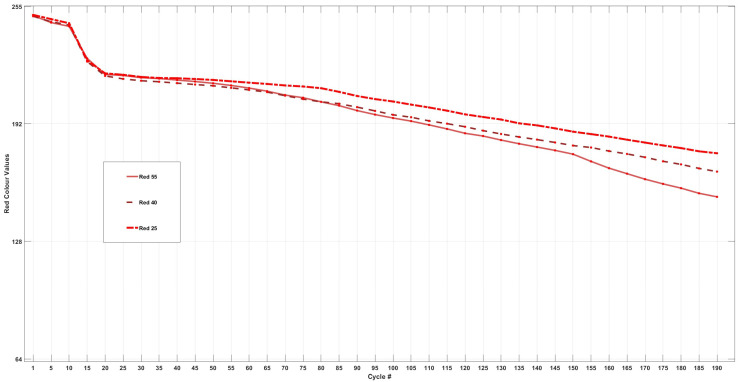
Change in red LED intensity for Cell55, Cell40, and Cell25.

Figure 11 shows the trends of each cell for the change in red intensity throughout 200 cycles.

The changes in red intensity for each cell are detailed in Table 7. According to the data, Cell55 experienced a total decrease of 39% over 200 cycles, representing the highest reduction in red intensity among the three cells and following similar trends to those observed in green and blue intensities. When comparing the percentage change in intensity between the first 100 cycles and the last 100 cycles, it is evident that Cell25 and Cell40 exhibited a 3% slower change in intensity during the latter half of the cycling period. In contrast, Cell55 experienced an increase of 3% in percentage change during the last 100 cycles. This observation may suggest the formation of specific species or chemical/side reactions taking place only at 55 °C, which in turn accelerate the drop in red intensity, indicating a greater reflectivity to red wavelengths at this temperature.

**Figure 12:**
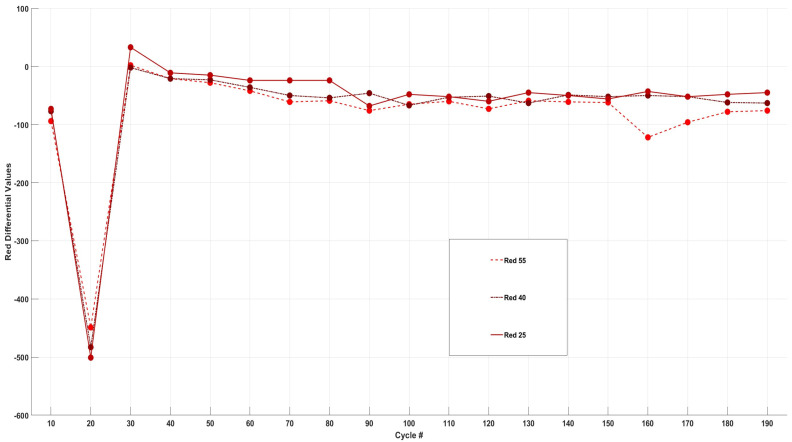
Differential analysis of change in red intensity for Cell55, Cell40, and Cell25.

Analysis of the differential data for the red intensities presented in Figure 12 reveals a consistent decline in red intensity across all three cells at the 20th cycle. A closer examination of the differential analysis indicates that the red intensity graph demonstrates a more synchronised change compared to the patterns observed in green and blue intensities. This synchronisation may suggest that the chemical/side reactions or the species formed during the cycling process exhibit a lower reflectivity to red wavelengths.

Upon further observation of the differential graph, notable peaks are evident at the 90th and 120th cycles for all three cells. Additionally, a sharp peak is uniquely observed for Cell55 at the 160th cycle. This peak can be correlated with the bar graph in Figure 5, which indicates that the electrolyte began to transition to its darkest shade of brown around this cycle, in comparison to Cell25 and Cell40. These findings underscore the distinct behaviour of red intensity relative to the other colour components throughout the cycling process. Overall, in comparison to the changes in green and blue intensities across the three cells, red intensity exhibited the lowest degree of colour change.

## 4. Conclusions

This study utilises an in situ optical sensing technique to detect electrolyte colour change demonstrating a relationship between high-temperature cycling, capacity change, and electrolyte colour. A comprehensive comparison of capacity loss among the three cells indicated that Cell55 experienced the highest reduction at 9%, while Cell25 showed the lowest at 6%. This trend supports the understanding that elevated temperatures lead to increased capacity loss, as expected.

The subsequent electrolyte colour analysis further illustrates a clear relationship between temperature and electrolyte colour change. Cell55, operating at the highest temperature (55 °C), displayed the darkest colour alteration, followed by Cell40, with Cell25 exhibiting the slightest change. This correlation indicates that the electrolyte colour has a direct correlation with temperature, with higher temperature cells resulting in more pronounced darkening.

To gain deeper insights into individual colour patterns, we conducted a detailed analysis of the individual colour intensities for each cell. The results indicated that Cell55 experienced the greatest changes across all three-colour intensities, with blue showing the most significant alteration for each cell and red exhibiting the least change. This pattern suggests that the species formed during cycling result in more colour change in blue wavelengths, followed by green. Notably, the change in blue intensity was particularly pronounced during the last 100 cycles, indicating that the chemical reactions species formation may be more reflective to blue during this period. In contrast, the change in green intensity remained uniform throughout the 200 cycles, while red intensity showed a slow decline of 3% for Cell25 and Cell40, coupled with a 3% increase for Cell55. Future research should correlate chemical reactions with electrolyte stability and performance which could suggest chemical/side reactions or specific species formation, i.e., species which are more reflective to red and occur predominantly at 55 °C after the initial 100 cycles.

To have a more detailed understanding of these events, a detailed chemical analysis is essential for elucidating specific degradation mechanisms, which will subsequently enhance our understanding of electrolyte breakdown and its relationship with colour change, which can be achieved using techniques such as FTIR, UV–VIS, and NMR spectroscopies. This improved understanding will contribute to the optimisation of electrolyte formulations and provide a novel and comprehensive insight into the processes governing electrolyte stability and performance.

## Figures and Tables

**Table 1:** Capacity fade percentage for Cell55, Cell40, and Cell25.

	% Change: 0–200 Cycles	% Change: 0–100 Cycles	% Change: 100–200 Cycles
Cell55	9%	6%	3%
Cell40	8%	5%	3%
Cell25	6%	4%	2%

**Table 2:** RGB and capacity change for Cell25.

	% Change: 0–200 Cycles	% Change: 0–100 Cycles	% Change: 100–200 Cycles
Red	30%	18%	15%
Green	42%	24%	24%
Blue	53%	28%	34%
Capacity	6%	4%	2%

**Table 3:** RGB and capacity change for Cell40.

	% Change: 0–200 Cycles	% Change: 0–100 Cycles	% Change: 100–200 Cycles
Red	35%	20%	19%
Green	47%	27%	28%
Blue	58%	33%	38%
Capacity	8%	5%	3%

**Table 4:** RGB and capacity change for Cell55.

	% Change: 0–200 Cycles	% Change: 0–100 Cycles	% Change: 100–200 Cycles
Red	39%	21%	24%
Green	51%	28%	31%
Blue	62%	34%	43%
Capacity	9%	6%	3%

**Table 5:** Change comparison of blue intensity between cells.

	% Change: 0–200 Cycles	% Change: 0–100 Cycles	% Change: 100–200 Cycles
Cell55	62%	34%	43%
Cell40	58%	33%	38%
Cell25	53%	28%	34%

**Table 6:** Change comparison of green intensity between cells.

	% Change: 0–200 Cycles	% Change: 0–100 Cycles	% Change: 100–200 Cycles
Cell55	51%	28%	31%
Cell40	47%	27%	28%
Cell25	42%	24%	24%

**Table 7:** Change comparison of red intensity between cells.

	% Change: 0–200 Cycles	% Change: 0–100 Cycles	% Change: 100–200 Cycles
Cell55	39%	21%	24%
Cell40	34%	20%	17%
Cell25	30%	18%	15%

## Data Availability

The datasets presented in this article are not readily available because the data are part of an ongoing study. Requests to access the datasets should be directed to Saud Sattar.
